# Detection of Honeybee Viruses in *Vespa orientalis*

**DOI:** 10.3389/fcimb.2022.896932

**Published:** 2022-05-04

**Authors:** Karen Power, Gennaro Altamura, Manuela Martano, Paola Maiolino

**Affiliations:** Department of Veterinary Medicine and Animal Productions, University of Naples “Federico II”, Naples, Italy

**Keywords:** ABPV, BQCV, DWV, spillover, Vespa

## Abstract

The Oriental hornet (*Vespa orientalis*) is spreading across the Italian territory threatening the health and wellbeing of honeybees by feeding on adult individuals and larvae and by plundering hive resources. Considering the capacity of other hornets in harboring honeybee viruses, the aim of this study was to identify the possible role of the Oriental hornet as a vector for honeybee viruses. Adult hornets were subjected to macroscopical examination to identify the presence of lesions, and to biomolecular investigation to detect the presence of six honeybee viruses: Acute Bee Paralysis Virus (ABPV), Black Queen Cell Virus (BQCV), Chronic Bee Paralysis Virus (CBPV), Deformed Wing Virus (DWV), Kashmir Bee Virus (KBV), Sac Brood Virus (SBV). No macroscopical alterations were found while biomolecular results showed that DWV was the most detected virus (25/30), followed by ABPV (19/30), BQCV (13/30), KBV (1/30) and SBV (1/30). No sample was found positive for CBPV. In 20/30 samples several co-infections were identified. The most frequent (17/30) was the association between DWV and ABPV, often associated to BQCV (9/17). One sample (1/30) showed the presence of four different viruses namely DWV, ABPV, BQCV and KBV. The detected viruses are the most widespread in apiaries across the Italian territory suggesting the possible passage from honeybees to *V. orientalis*, by predation of infected adult honeybees and larvae, and cannibalization of their carcasses. However, to date, it is still not clear if these viruses are replicative but we can suggest a role as mechanical vector of *V. orientalis* in spreading these viruses.

## Introduction

Among the arthropods which can be identified as natural enemies of *Apis mellifera* (Linnaeus, 1758), social hornets of the Vespidae family appear to be most dangerous for the survival of the honeybee colony, sometimes leading it to collapse (Matsuura, 1988; Matsuura and Yamane, 1990; Laurino et al., 2020). Three main species of Vespa have been identified on the Italian territory: the European hornet (*Vespa crabro* Linnaeus, 1761) (Carpenter and Kojima, 1997), the Oriental hornet (*Vespa orientalis* Linnaeus, 1771) (Ćetković, 2003) and the Asian hornet (*Vespa velutina* Lepeletier, 1836) (Bertolino et al., 2016). While the first is considered as native species in Italy, the Oriental hornet and the Asian hornet are invasive species which are widely spreading in the Italian territory (Laurino et al., 2020; Graziani and Cianferoni, 2021). The Oriental hornet is native to the south-eastern Mediterranean, north-eastern and eastern Africa, the Middle East, Central Asia (Archer, 1998; Ćetković, 2003), but it has expanded its areal due to involuntary anthropic introduction and to climate change (Ward and Masters, 2007; Renault et al., 2018) and habitat loss, leading to novel combinations of interacting species. In fact, it has been reported that there is an alarming association between the rise of temperatures and the settlement of species outside their natural range, particularly for terrestrial arthropods (Hulme, 2017). In 2020, populations of Oriental hornets have been reported also in Chile (Ríos et al, 2020), while they were previously spotted in Mexico and Brasil (du Buysson, 1904; Dvořák, 2006). In Italy the Oriental hornet is well established in Sicily, Calabria, Lazio and Campania regions, however new reports have recorded it also in Liguria (Gereys et al., 2021), Trieste (Bressi et al., 2019) and more recently in Tuscany (Graziani and Cianferoni, 2021). *Vespa orientalis* is distinguishable from *V. crabro* and *V. velutina* as it is characterized by a rusty red color, yellow marks on the head between the eyes and yellow bands in the abdominal metasoma (Linnaeus, 1771; Smith-Pardo et al., 2020). Oriental hornets live in seasonal colonies, which originate by a single queen which emerges in spring after a period of hibernation. Although the queen’s main role is to lay eggs in the new colony and enlarge the nest by building new brood comb, she can visit hives to collect food for the developing brood of the “embryonic nest” (Matsuura, 1991). After approximately a month, workers emerge and the colony develops throughout spring and summer reaching a peak of thousands of individuals in late summer/beginning of fall. Workers attend to the activities previously performed by the queen while she continues with her main role of oviposition; therefore, workers are busy providing food for the brood, cleaning and enlarging nest (Cappa et al., 2021). Between September and December the colony population drops, meanwhile new queens and drones enclose and soon mate. Soon after, while drones die, fertilized queens go in search of a dark and dry hideout for the winter diapause (Ishay, 1964). Adult hornets feed mainly on carbohydrates which are taken up by ingestion of mature fruit, such as apples, dates and grapes (Ibrahim and Mazeed, 1967), nectar and honey, while the brood is fed mainly animal proteins (grasshoppers, flies, yellowjackets and honeybees) (Archer, 1998; Hernández et al., 2013). In order to grow and develop correctly, hornet larvae need great amounts of proteins, and honeybees are the favored prey among the other animal and non-animal protein sources (Cini et al., 2018). Forager hornets are able to locate and encircle honeybee hives which present the best combination of sugars and proteins, attacking honeybee foragers which are leaving for or coming back from the foraging activities (Ishay, 1964; Werenkraut et al., 2021) and plunder the hive by robbing and taking to their nests honey, pollen and larvae, leading to the weakening of honeybee colonies (Morse, 1978; Abrol, 1994). Besides the reduction in the number of honeybees due to predation, the number of workers in a colony can decrease as a result of starvation consequent to inhibition of *A. mellifera* foraging activity and scarcity of hive food storage, which is robbed by the hornets (Monceau et al., 2013, Monceau et al., 2014). Moreover, reduction of nectar and pollen intake, could result in poorly nourished larvae and weak adults, which are less resistant to pathogens and pesticides, eventually resulting in colony collapse (Branchiccela et al., 2019). In addition to the negative consequences on the beekeeping industry and on the ecosystem services associated to pollination by *A. mellifera*, the hornet-honeybee interaction can lead to the passage of pathogens between the species, and hornets can act as vectors for already known honeybee pathogens and as potential spillover agents for new pathogens from invasive species to native species, as reported in the review by Lester and Beggs (2019). Previous studies have already detected the presence of various honeybee viruses (Forzan et al., 2017; Mazzei et al., 2018; Mazzei et al., 2019; Highfield et al., 2020), fungi (Gabín-García et al., 2021) and bacteria in social hornets (Nowar, 2016), underlining the possible role of these insects in spreading and transmitting pathogens which could be potentially lethal to honeybees. Among honeybee pathogens, viruses can be considered as cause of severe disease for honeybees threatening their health and well-being, often leading to the collapse of entire colonies (Brutscher et al., 2016). To date, little information is available on the presence of honeybee pathogens in *V. orientalis*. The aim of this study was to assess the presence of six honeybee viruses, Acute Bee Paralysis Virus (ABPV), Black Queen Cell Virus (BQCV), Chronic Bee Paralysis Virus (CBPV), Deformed Wing Virus (DWV), Kashmir Bee Virus (KBV), Sac Brood Virus (SBV), in samples of *V. orientalis*.

## Materials and Methods

In November 2021, 30 adult Oriental hornets were collected from three different apiaries located in the Campania region (A= 40°51’17.866”N, 14°22’14.671” E; B=40°50’32.898” N, 14°4’50.426 E; C=40°52’31.896” N, 14°18’26.154” E). Samples were captured during their predatory activity in the proximity of the hives using a butterfly net. All samples were transported in 50 mL tubes to the laboratory of Veterinary General Pathology and Anatomical Pathology of the Department of Veterinary Medicine and Animal Productions, University of Naples “Federico II” where they were immobilized with chilling for 5 min at -20°C.

**Figure 1:**
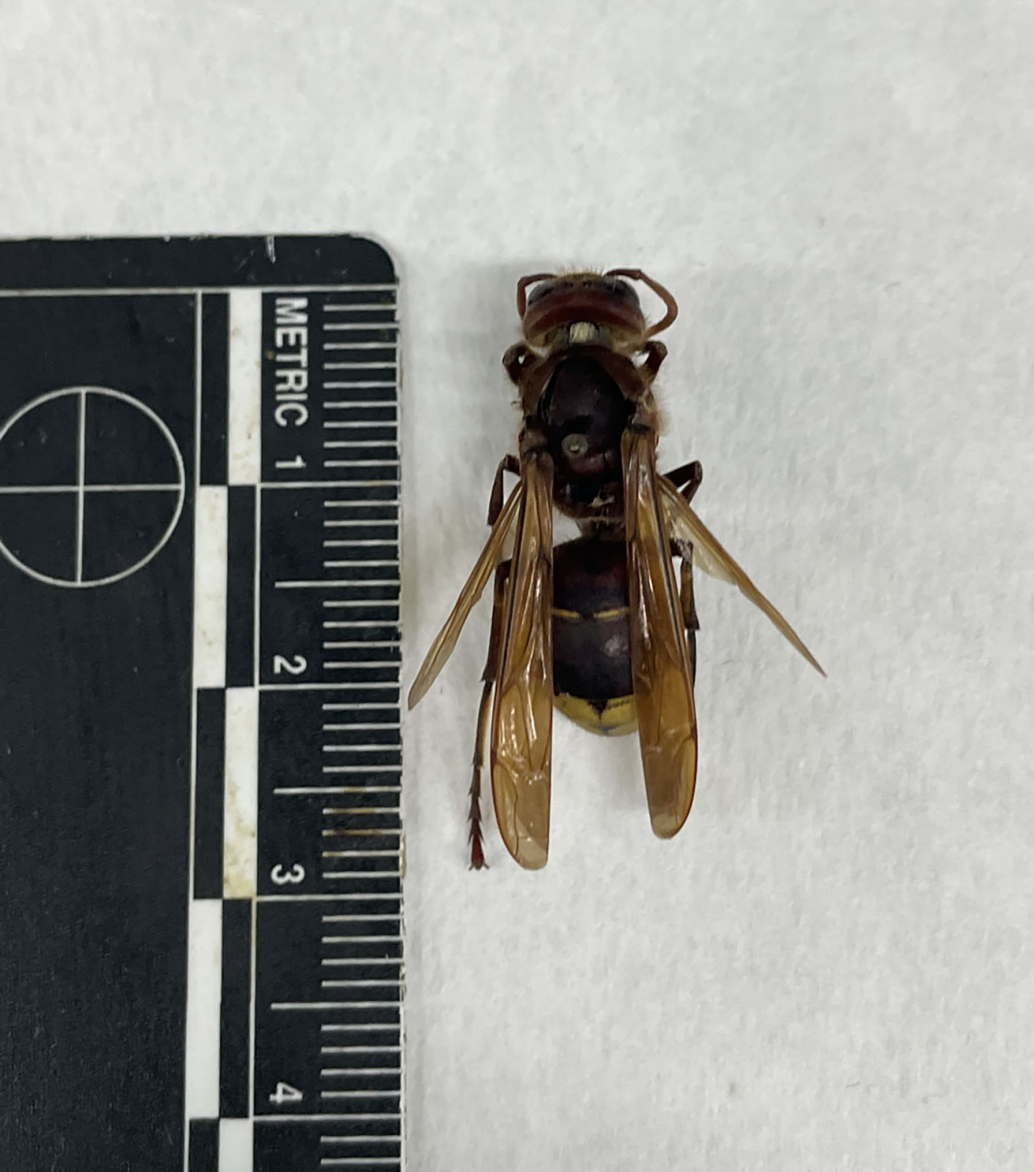
Adult individual of *Vespa orientalis* showing the typical yellow bands of the abdominal metasoma.

A total of 30 hornets (10 individuals/sampling site) were then subjected to macroscopical examination by observation at the stereo microscope (Microscope Axioskop HBO50, Zeiss, Milan, Italy) in order to identify possible visible alterations, namely crippled wings, shortened and discolored abdomens, which could be indicative of viral infections (**Figure 1**). The same samples where then subjected to molecular biology analysis to detect the possible presence of honeybee viruses. For each animal, head, legs and wings were removed while the thorax and the abdomen were cut into small pieces with a sterile blade to ease homogenization with the TissueLyser mechanical homogenizer (Qiagen, Hilden, Germany), as previously described (Power et al., 2021). RNA was extracted and purified from genomic DNA using the RNeasy Plus Mini Kit (Qiagen, Hilden, Germany), according to the protocol provided by the manufacturer, and subjected to reverse transcription (RT) PCR as reported elsewhere (Altamura et al., 2018). A multiplex PCR thermal protocol was executed to screen for the presence of six relevant honeybee viruses (ABPV, BQCV, CBPV, DWV, KBV, SBV) according to the procedure proposed and validated by Cagirgan and Yazici (2020). A 150 base pairs segment of 28s ribosomal RNA of *V. orientalis* was also amplified as a housekeeping gene to ensure the presence of amplifiable cDNA in each sample by using the following primer pair designed on the sequence available on GenBank (KF933071.1): *V.Orientalis* 28S_1 FW: TGGGATGAACCAAACGCAGA, *V.Orientalis* 28S_1 REV: CTAGTTGCTTCGGCAGGTGA. Moreover, in each PCR reaction, one no template control (NTC) was included as negative control. Honeybees’ samples harboring DWV, SBV, ABPV, BQCV and gBlocks Gene Fragments (Integrated DNA Technologies, Coralville, IA, USA) mimicking CBPV and KBV intended amplicons were employed as positive controls. Amplification products were then migrated by electrophoresis on 2.5% agarose gel in TAE buffer (Tris-Acetate-EDTA) along with a 100 bp molecular marker (Bioline), stained with ethidium bromide and observed under UV with the ChemiDoc gel scanner (Bio-Rad). Ethical review and approval were waived for this study, as according to the D.L. 4 March 2014 n.26, and national implementing decree following the European regulation 2010/63/UE, ethical approval is not necessary for invertebrates with the except of Cephalopoda.

## Results

Macroscopical examination did not reveal any alterations in the examined samples (30/30; 100%), which could have suggested the presence of viral infection, such as crippled wings, shortened and discolored abdomens. PCR results showed that 28/30 (93%) samples were positive for at least one virus, while no sample was found positive for CBPV. DWV was detected in 25/30 samples (83%), ABPV in 19/30 samples (63%) and BQCV in 13/30 samples (43%). KBV and SBV cDNAs were amplified in 1/30 samples (3%) and 1/30 samples (3%), respectively. 28s ribosomial RNA amplification confirmed the integrity of all analyzed cDNAs.

Moreover, several co-infections were identified in 20/30 (67%) samples. Among all, the association between DWV and ABPV was the most frequent (17/30; 56%), often associated to BQCV (9/17; 53%). One sample (1/30; 3%) showed the presence of four different viruses namely DWV, ABPV, BQCV and KBV.

For a comprehensive view of PCR positivity and additional co-infections, see **Table 1**.

**Table 1:** Detection of honeybee viruses and 28s ribosomal RNA in *V.orientalis* by multiplex PCR.

Apiary	Sample	DWV	SBV	ABPV	BQCV	KBV	CBPV	28S
A	VO1	+	–	+	+	–	–	+
	VO2	+	–	+	+	–	–	+
	VO3	+	–	+	+	–	–	+
	VO4	+	–	+	+	–	–	+
	VO5	+	–	+	+	+	–	+
	VO6	+	–	+	+	–	–	+
	VO7	–	–	+	+	–	–	+
	VO8	+	–	+	–	–	–	+
	VO9	+	–	–	+	–	–	+
	VO10	–	–	+	–	–	–	+
B	VO11	+	–	+	+	–	–	+
	VO12	+	–	+	+	–	–	+
	VO13	–	–	–	–	–	–	+
	VO14	+	–	+	–	–	–	+
	VO15	+	–	–	–	–	–	+
	VO16	–	–	–	+	–	–	+
	VO17	+	–	+	–	–	–	+
	VO18	+	–	+	–	–	–	+
	VO19	+	–	+	–	–	–	+
	VO20	+	+	+	–	–	–	+
C	VO21	+	–	–	–	–	–	+
	VO22	+	–	–	+	–	–	+
	VO23	+	–	–	–	–	–	+
	VO24	+	–	+	+	–	–	+
	VO25	–	–	–	–	–	–	+
	VO26	+	–	–	–	–	–	+
	VO27	+	–	+	–	–	–	+
	VO28	+	–	–	–	–	–	+
	VO29	+	–	–	–	–	–	+
	VO30	+	–	+	–	–	–	+

Sampling apiary, sample names and viruses acronyms are indicated (DWV, Deformed Wing Virus; SBV, SacBrood Virus; ABPV, Acute Paralysis Virus; BQCV, Black Queen Cell Virus; KBV, Kashmir Bee Virus; Chronic Bee Paralysis Virus; + positive sample; - negative sample).

## Discussion

Honeybees can be threatened by a great number of pathogens, many of which have been encountered also in other arthropods (Eyer et al., 2009; Martin and Brettell, 2019; Gusachenko et al., 2020; Payne et al., 2020). Spillover from honeybees to other managed or wild species (Pritchard et al., 2021), and from other species to honeybees, has been widely reported and the number of described events has grown during the past decades due to increasing interest of the scientific community in understanding honeybee pathogen prevalence and epidemiology (Tehel et al., 2016; Gisder and Genersch, 2017; Yañez et al., 2020; Dalmon et al., 2021).

To date many viruses which can infect honeybees have been reported and the list is continuously increasing (Tantillo et al., 2015; Grozinger and Flenniken, 2019), also due to spillover events. For instance, the Moku virus (MV), a novel Iflavirus closely related to the Slow Bee Paralysis Virus, was discovered in the social wasp *Vespula pensylvanica*, but MV was also detected in honeybees, suggesting a possible passage from the wild wasp to domesticated honeybees (Mordecai et al., 2016). Social hornets pertaining to the *Vespa* genus are spreading across the Italian territory causing great damage to the beekeeping field by predating honeybee foragers and larvae, pillaging honeybee food supplies and participating to the diffusion of pathogens. Previous studies have reported the presence of replicative forms of DWV (Mazzei et al., 2018), BQCV and KBV in *Vespa velutina* (Mazzei et al., 2019), as well as DWV in *Vespa crabro* (Forzan et al., 2017). It has already been shown that *Vespa orientalis* can be a vector of *Paenibacillus larvae*, causative agent of the American Foulbrood (Nowar, 2016). This study describes the presence of honeybee viruses in *V. orientalis*. Biomolecular analysis showed that, in the specimens analyzed, DWV was the most prevalent virus (83%), followed by ABPV (63%) and BQCV (43%), while KBV and SBV were present only in one sample each. No other virus was identified in our samples. Our results are in line with previous data, in fact, among the viruses most frequently found in non-honeybee arthropods, DWV is the most detected, while BQCV, ABPV, SBV and KBV result highly present, however with lower frequency (Nanetti et al., 2021). Moreover, in our specimens the presence of co-infection was found. Co-infections have already been described in honeybees (Evans, 2001), as well as in *V.velutina*, were KBV and BQCV were found in the same individuals (Mazzei et al., 2019). Conversely to what previously reported in other species (Genersch et al., 2006; Forzan et al., 2017), none of our samples showed any lesions at the macroscopical examination, indicative of the presence of viruses. However, as demonstrated by a previous study, we cannot exclude that, as in honeybees, viruses are generating damage to tissues in asymptomatic individuals (Power et al., 2021). In honeybees, common sources of viral infections are pollen, nectar, bee bread, royal jelly and honey (Mazzei et al., 2014; Schittny et al., 2020) as well as transmission mediated by *Varroa destructor* (Shen et al., 2005; Santillán-Galicia et al., 2010). According to the information known about the biology of *V.orientalis*, we can very likely exclude possible infection of the hornet by pollination activity (i.e. contact with infected pollen) and parasite infestation, and suppose that they become infected by predating infected adult honeybees and larvae, and cannibalizing their carcasses. As a matter of fact, the viruses found in our hornet samples are also the most widespread in the apiaries across the Italian territory (Porrini et al., 2016; Bellucci et al., 2019) and the Campania region (Maiolino et al., 2022), suggesting a possible passage from honeybees to *V. orientalis*. However, as honeybee viruses can be transmitted *via* honey (Mutinelli, 2011) we should also consider possible transmission through ingestion of honey, or conspecific feeding in close proximity of rotten fruits or flowers. Moreover, to date, it is still not clear if these viruses are replicative and represent real infections or if the Oriental hornet is an “incidental host” which acquired viruses passively through feeding. Future analysis aimed at quantifying the viral load and sequencing of the viral genetic material harbored by both Oriental hornet and honeybees collected from the same apiary, could help to fill these gaps. Further studies are needed to better understand the role of *V. orientalis* in spreading these viruses.

## Data Availability Statement

The original contributions presented in the study are included in the article/supplementary material. Further inquiries can be directed to the corresponding author.

## Author Contributions

Conceptualization, KP and PM; Methodology, KP and GA; Validation, MM and PM; Writing—Original Draft Preparation, KP; Writing—Review and Editing, MM and PM; Supervision, MM and PM. All authors read and approved the final version of this manuscript.

## Funding

The authors received financial support from the research funding of the Department of Veterinary Medicine and Animal Productions, University of Naples "Federico II" Italy.

## Conflict of Interest

The authors declare that the research was conducted in the absence of any commercial or financial relationships that could be construed as a potential conflict of interest.

## Publisher’s Note

All claims expressed in this article are solely those of the authors and do not necessarily represent those of their affiliated organizations, or those of the publisher, the editors and the reviewers. Any product that may be evaluated in this article, or claim that may be made by its manufacturer, is not guaranteed or endorsed by the publisher.
